# Preparation of freeze‐dried bioluminescent bacteria and their application in the detection of acute toxicity of bisphenol A and heavy metals

**DOI:** 10.1002/fsn3.2800

**Published:** 2022-04-11

**Authors:** Jun Yang, Shulin Hu, Anqi Liao, Yetian Weng, Shuli Liang, Ying Lin

**Affiliations:** ^1^ 26467 School of Biology and Biological Engineering South China University of Technology Guangzhou China; ^2^ 26467 Guangdong Provincial Key Laboratory of Fermentation and Enzyme Engineering South China University of Technology Guangzhou China

**Keywords:** acute toxicity, bioluminescent bacteria, BPA, heavy metal, *Vibrio qinghaiensis* Q67

## Abstract

Current chemical analysis approaches for contaminants have failed to reveal their biotoxicity. Moreover, conventional bioassays are time consuming and exhibit poor repeatability. In this study, we performed the acute toxicity detection of various contaminants (chromium (Cr), cadmium (Cd), lead (Pb), arsenic (As), mercury (Hg), tin (Sn), nickel (Ni), and bisphenol A (BPA)) with four bioluminescent bacteria (*Vibrio qinghaiensis* Q67, *V. fischeri*, *Photobacterium phosphoreum* T3, and *P. phosphoreum* 502) using a rapid, flexible, and low‐cost bioassay. We found that the temperature affected the bacterial luminescence, and freeze‐dried cells exhibited sensitive toxic responses to contaminants. Indeed, the optimized protectants containing 12% (w/v) trehalose, 4% sucrose, and 2% sorbitol displayed better luminescence and toxic sensitivity. Furthermore, freeze‐dried powders of these strains were prepared and subjected to acute toxicity detection. The results showed that all contaminants exhibited acute toxicity toward Q67, but the other strains did not show obvious response to nickel and tin. The relative half‐maximal effective concentration (EC_50_) values of BPA, Cr, Cd, Pb, As, Hg, Ni, and Sn to Q67 were 0.674, 1.313, 11.137, 5.921, 4.674, 0.911, 5.941, and 54.077 mg/L, respectively. In addition, the EC_50_ values of contaminants toward different strains were suggested to be statistically significant. Freeze‐dried Q67 exhibited toxic responses to more contaminants than the other bioluminescent strains; therefore, Q67 was selected to be more suitable than the other strains for single and mixture toxicity detection tests. Compared with other strains, Q67 was more appropriate for the rapid screening of the mixture toxicity of contaminants in samples as a nonspecific screening sensor before the use of standard analysis approaches.

## Graphical abstract

This work compared the difference in the acute toxicity of BPA and heavy metals toward four natural bioluminescent bacteria. Freeze‐dried *Vibrio qinghaiensis* Q67 revealed toxic respond to all contaminants, but other strains failed to respond to some contaminant. The EC50 of all contaminants toward different bioluminescent strains suggested to be statistically significant, which indicated *V. qinghaiensis* Q67 exhibited more application potential than other strains in single and mixture toxicity detection.

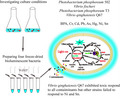

## 1 INTRODUCTION

Heavy metals and bisphenol A (BPA) are widely spread in the environment and also found in food and food packaging materials. BPA is an industrial chemical that is widely used as a monomer of polycarbonate, an antioxidant of polyvinyl chloride (PVC), in food contact materials, such as plastic bottles, cups, plates, goblets, and storage containers (Geens et al., 2012). The increasing demand for BPA has resulted in its accumulation in the environment (P. Chen et al., 2021; Huang et al., 2012; Yang et al., 2020). In some region with pollution of BPA, the concentration of BPA has reached 174.6 μg/ml in the river (Ronderos‐Lara et al., 2018) and 25 mg/L BPA in the landfill leachate (Schwarzbauer et al., 2002). Consequently, BPA pollution has become an increasingly serious issue. Heavy metals and BPA present in food or transferred to food via food packaging materials get released into the human body through daily food intake. BPA exhibits obvious reproductive, immune, developmental, endocrine, and nervous system toxicity (Gowder, 2013). Meanwhile, heavy metals, such as chromium (Cr), cadmium (Cd), lead (Pb), arsenic (As), mercury (Hg), tin (Sn), and nickel (Ni), can accumulate in the human body, resulting in various diseases of the target organs (Mansour, 2014). Therefore, detection of these contaminants is crucial. Atomic absorption spectroscopy, gas chromatography (GC), high‐performance liquid chromatography (HPLC), mass spectrometry (MS), and various combined chromatography‐mass spectrometric techniques (such as GC‐MS and LC‐MS) are used as the standard analysis methods for such contaminants. However, the pretreatment process of samples as well as the analysis steps are complicated and the equipment used is expensive. Moreover, these methods cannot effectively reveal the toxicity of the sample (Gomes et al., 2017; Jia & Ionescu, 2016; Le et al., 2017). Additionally, conventional bioassays using animals, plants, zebrafish, and other aquatic or terrestrial organisms are time consuming, have poor repeatability, high cost, and are resistant to the 3R principles (replacement, reduction, and refinement) for the ethical use of animals in testing (Abbas et al., 2018; Kudryasheva & Tarasova, 2015; Riebeling et al., 2018). Compared with these methods, bioluminescent bacterial detection is a rapid and low‐cost bioassay that exhibits several advantages, such as flexible testing, high sensitivity, real‐time monitoring, less demand for samples, and low consumption (Bolelli et al., 2016; Pivato & Gaspari, 2006). Natural bioluminescent bacteria are commonly used to measure the acute toxicity of contaminants (Jia & Ionescu, 2016; Thouand & Marks, 2014). Natural bioluminescent bacteria are mainly observed in four genera, namely *Photobacterium*, *Photorhabdus*, *Shewanella*, and *Vibrio*, and most of them have been isolated from the ocean (Caccamo et al., 1999; G. Thouand & Marks, 2016). *Photobacterium phosphoreum* T3, *P*. *phosphoreum* 502, *Vibrio fischeri* (NRRL B‐11177), and *V*. *qinghaiensis* Q67 have been studied to date (Gérald Thouand & Marks, 2014; G. Thouand & Marks, 2016). *V*. *qinghaiensis* Q67 is widely used for acute and mixture toxicity detection as it is the only freshwater‐isolated strain, and hence, there is no requirement to add high concentrations of NaCl to test the samples (Ma et al., 1999; Rodea‐Palomares et al., 2009; Wu et al., 2018; Xu et al., 2020; Xu et al., 2018; Zhang et al., 2018, 2020), which is beneficial as high concentrations of NaCl are known to influence the toxicity of the contaminant (Ankley et al., 1989; Hinwood et al., 1987; Rodea‐Palomares et al., 2009).

**FIGURE 1:**
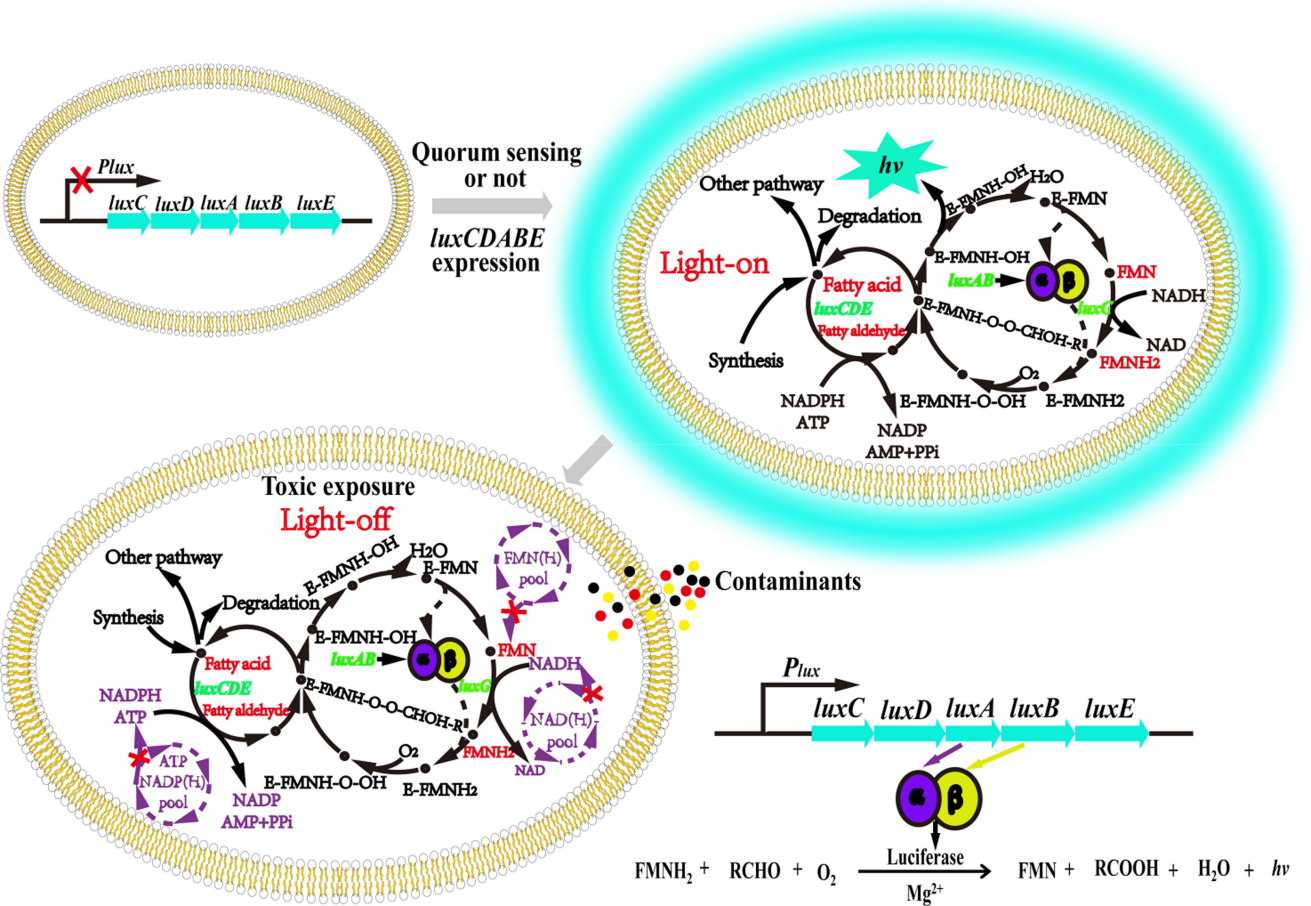
Light emission and decrease in natural bioluminescence in bacteria. Transcription of genes *luxCDABE* was triggered when the cell mass reached a threshold concentration in *Vibrio fischeri* and *V. qinghaiensis* Q67, while luminescence was not triggered by this mechanism in the other strains

Bioluminescence of natural bioluminescent bacteria is due to the light emission via a luciferase‐catalyzed reaction (Wilson & Hastings, 1998). Light emission occurs when the *lux* operon is transcribed and translated in luminescent bacteria, which may be relative to quorum sensing or not (Flodgaard et al., 2005; Fuqua et al., 1994; Gray et al., 1994; Tanet et al., 2019). The *lux* operon mainly contains *luxCDABEG* and an extra *luxF* gene in some strains, such as the members of *Photobacterium*. The gene *luxG* has been proposed to be a flavin reductase that generates the coenzyme FMNH_2_ (Nijvipakul et al., 2008). Genes *luxA* and *luxB* express the α and β subunits of luciferase, respectively (Fisher et al., 1996; Suadee et al., 2007). Genes *luxC*, *luxD*, and *luxE* express reductase, transferase, and synthetase, respectively. The three enzymes combine to form a fatty acid reductase multienzyme complex that catalyzes the long‐chain fatty aldehyde synthesis reaction (Meighen, 1991; Sitnikov et al., 1995). In the presence of oxygen and cofactors (such as NADH, NADPH, ATP, and Mg^2+^), FMNH_2_ and long‐chain aldehydes were catalyzed into FMN, fatty acid, and water with blue‐green light (450–490 nm) emission (Figure 1). Toxicity bioassays using bioluminescent bacteria are based on the fact that the light emission of bacteria decreases in the presence of contaminants. The contaminant impairs the intracellular metabolism and high‐energy cofactor supplementation for a short period of time (Bulich & Isenberg, 1981; Gérald Thouand & Marks, 2014) (Figure 1). The reduction in light emission with increasing contaminant concentration as well as the toxicity of the contaminants were described by relative half‐maximal effective concentration (EC_50_) values of the contaminants. The contaminant causes a 50% decrease in the original light when its concentration is EC_50_ (Bulich & Isenberg, 1981).

The acute toxicity of metals and other toxic contaminants in aquatic samples using bioluminescent bacteria has been well studied. The acute toxicity of the same contaminant to different bioluminescent bacteria appears to be different (Gérald Thouand & Marks, 2014). In addition, the application of acute toxicity detection for contaminants in food is limited as the complex components in food affect the toxicity of the target contaminant. Therefore, we measured the total mixture toxicity of the contaminants in food samples (Gérald Thouand & Marks, 2014; G. Thouand & Marks, 2016). There are some studies on the acute toxicity of specific or nonspecific contaminants in food (Alocija & McLean, 2007; Pellinen et al., 2002; Wu et al., 2018). This study focuses on simple samples, such as water. Detection of acute toxicity in other samples, such as milk or food, is suggested to be non‐specific mixture toxicity detection. Therefore, a bioluminescent bacterial strain that sensitively responds to many contaminants is suitable for detecting the toxicity of food or environmental samples. In this study, the acute toxicity of heavy metals and BPA was investigated using four freeze‐dried natural bioluminescent bacteria, and the differences in the acute toxicities of the contaminants toward the four bioluminescent bacteria were compared. In fact, studies using the freeze‐dried powder preparations of these bioluminescent bacteria, especially Q67, are lacking. Furthermore, the acute toxicities of aqueous BPA solution (organic solvent free), Ni, and Sn were tested using bioluminescent bacteria for the first time in this study. Optimized protectants containing 12% trehalose, 4% sucrose, and 2% sorbitol were obtained by response surface analysis. The results revealed that *V*. *qinghaiensis* Q67 exhibited sensitive toxic responses to all contaminants used in this study, but the other strains failed to respond to some contaminants. Moreover, the acute toxicity of BPA was stronger than that of all other contaminants used in this study.

## 2 MATERIALS AND METHODS

### 2.1 Bacterial strains

All bioluminescent bacterial strains used in this study were *P*. *phosphoreum* T3, *V*. *fischeri*, *P*. *phosphoreum* 502, and *V*. *qinghaiensis* Q67. *P*. *phospohoreum* T3 was purchased from the Institute of Soil Science, Chinese Academy of Sciences. *V*. *fischeri* was purchased from the China Center of Industrial Culture Collection (CICC 10,483, also known as NRRL B‐11177). *P*. *phosphoreum* 502 was presented by Professor Weitie Lin from the South China University of Technology. *V*. *qinghaiensis* Q67, deposited in the China Center for Type Culture Collection (CCTCC No. M2010104), was kindly provided by Professor Jin Zhang from the Anhui University of Architecture.

### 2.2 Reagents and contaminants

Trehalose, sucrose, monosodium glutamate, sorbitol, and NaCl were purchased from Aladdin (Shanghai, China). The contaminants used in this study were BPA, K_2_Cr_2_O_7_, Cd(NO_3_)_2_, ZnSO_4_.7H_2_O, Pb(NO_3_)_2_, As, Hg, Ni, and Sn. BPA (GC, purity >99%) was purchased from Macklin (Shanghai, China). All other chemicals were purchased from Aladdin (Shanghai, China). As (analytical standard, 1000 μg/ml in 1.0 mol/L HNO_3_), Hg (analytical standard, 1000 μg/ml in 1.0 mol/L HNO_3_), Ni (100 μg/ml in 1% HNO_3_), and Sn (1000 μg/ml in 3.0 mol/L HCl with trace of HNO_3_) were used as standard solutions. All these chemicals were prepared in a solution containing 0.85 or 2% NaCl (w/v), and the pH values of the solutions were adjusted to 6.0–7.0. The concentration of these contaminants ranged from 0.02 to 100 mg/L.

### 2.3 Culture medium and culture conditions

The culture medium of *P*. *phosphoreum* T3 and 502 consisted of tryptone (5 g/L), yeast extract (5 g/L), KH_2_PO_4_ (1 g/L), Na_2_HPO_4_ (5 g/L), NaCl (30 g/L), and glycerol (3 ml/L), and pH was adjusted to 7.0 using HCl and NaOH solutions. The culture medium of *V*. *fischeri* consisted of tryptone (5 g/L), yeast extract (0.5 g/L), NaH_2_PO_4_.H_2_O (6.1 g/L), K_2_HPO_4_.3H_2_O (2.75 g/L), MgSO_4_.7H_2_O 0.204 (g/L), (NH_4_)_2_HPO_4_ (0.5 g/L), NaCl (30 g/L), and glycerin (3 ml/L), and pH was adjusted to 7.0. The culture medium of *V*. *qinghaiensis* Q67 consisted of tryptone (5 g/L), yeast extract (5 g/L), MgCl_2_ (3.2 g/L), KBr (0.2 g/L), CaSO_4_ (0.1 g/L), KCl (4 g/L), NaCl (4 g/L), and glycerol (3 ml/L), and pH was adjusted to 8.5 (Zhu et al., 2009). For solid culture medium, 1.5%–2% (w/v) agar was added. All culture media were sterilized at 121°C for 20 min. These bioluminescent strains were cultured at 20, 23, and 27°C with or without shaking at 180 rpm.

### 2.4 Preparation of bioluminescent bacteria used for acute toxicity detection

Bioluminescent bacterial strains were first cultured on a plate for 48–72 hr. A single colony was transferred into a test tube containing 5 ml medium and then cultured at the corresponding temperature for 14, 16, or 20 hr with shaking at 180 rpm. Then, the fresh culture was transferred into a 250‐ml flask containing 50 ml medium and cultured under the same conditions described previously. Finally, the culture was transferred into another 250‐ml flask with an initial optical density (OD)_600_ of 0.02, and then cultured under the same conditions to prepare the luminescent bacteria used for acute toxicity detection.

The bioluminescent bacteria used for acute toxicity detection was prepared using three methods. Method 1: Fresh culture was directly used for acute toxicity detection. Method 2: Fresh culture was centrifuged at  8228 *g* at 4°C for 10 min to collect the cells, which were then washed with NaCl solution and centrifuged under the same conditions. Finally, the cleaned cells were suspended in a protectant according to the international standard (ISO11348‐1) (8 g/L C_6_H_12_O_6_·H_2_O, 20 or 8.5 g/L NaCl, 2.035 g/L MgCl_2_.6H_2_O, 0.3 g/L KCl, 11.9 g/L HEPES, pH 7.0). Method 3: Fresh cultured cells were collected and washed, then suspended in a protectant. Finally, the bacterial suspension was freeze‐dried. To prepare the freeze‐dried luminescent cells, the fresh cells harvested from the 50 ml culture were suspended in a 2.5 ml protectant solution containing trehalose, sucrose, sorbitol, or monosodium glutamate. Then, 100 μL of suspended cells were transferred into 2‐ml round‐bottom centrifuge tubes at room temperature for 15 min and the centrifuge tubes were stored at –80°C for 10 h and freeze‐dried at –80°C for 12 h. The freeze‐dried cells were resuscitated in 0.85 or 2% (w/v) NaCl solution at room temperature for 15 min, and then used for the acute toxicity test.

### 2.5 Analytical procedures

A multifunctional microplate reader (TECAN Infinite M200, Switzerland) was used to measure the bacterial biomass and luminescence. The biomass, which is characterized at OD_600_, was tested using an absorbance module. As the luminescence intensity of these bacteria was beyond the measurement range of this instrument when using a 96‐well flat‐bottom white plate, a 96‐well flat‐bottom black plate was used instead.

For the acute toxicity test, 150 μl of the contaminant solution (150 μl of 0.85 or 2% NaCl solution was used as a control) was added to a 96‐well flat‐bottom white plate and then 10 μL of fresh prepared luminescent bacteria was added. After 15 min of incubation, luminescence intensity was detected. The inhibition of luminescence indicates the toxicity of the contaminant, which was calculated using Equation 1. Furthermore, the concentration–inhibition data were fitted by the logistic dose–response model (Backhaus et al., 1997; Chen et al., 2020). The acute toxicity of these contaminants was described by the EC_50_ value, which was calculated using a fitting function.

(1) $$I=\frac{Lc‐Ls}{Lc}\;\times \;100\%$$

where *I* is the inhibition ratio of luminescence. *L*c and *L*s indicate the relative luminescence units of luminescent bacteria exposed to the control and contaminant chemical solutions for 15 min, respectively.

### 2.6 Statistical analysis

Statistical analysis was conducted using IBM SPSS software (version 26.0). The differences in EC_50_ values between the four bioluminescent strains toward the same contaminant were analyzed. Statistical significance was set at *p* < .05. The quality of the developed fitting model was evaluated using coefficients (*R* ^2^).

## 3 RESULTS

### 3.1 Culture conditions of bioluminescent bacteria

The growth and luminescence of bioluminescent bacteria are mainly influenced by pH, temperature, and dissolved oxygen during cultivation. Despite the oxygen participating in the luminescence reaction, luminescence was strong at 180 rpm shaking culture according to previous studies. In this study, the impact of different shaking rates (140, 180, and 220 rpm) on cell growth and luminescence was investigated, but no obvious differences were observed in cell growth. Furthermore, the luminescence intensities were 2.3, 3.1, and 3.3 × 10^5^ units at 140, 180, and 220 rpm after 16 h of cultivation, respectively. Hence, the shaking rate was confirmed at 180 rpm, according to reference. In addition, a previous study indicated that the extracellular medium pH had less influence on luminescent bacteria (Ma et al., 1999). In this work, the impact of different pH values (7.5, 8.0, 8.5, and 9.0) on the cell growth and luminescence of *V*. *qinghaiensis* Q67 was investigated, and the luminescence intensities were 1.7, 1.4, 1.6, and 1.9 × 10^5^ units after 12 h of cultivation, respectively. Considering the small difference, the pH of the medium was consistent with the value reported in references.

**FIGURE 2:**
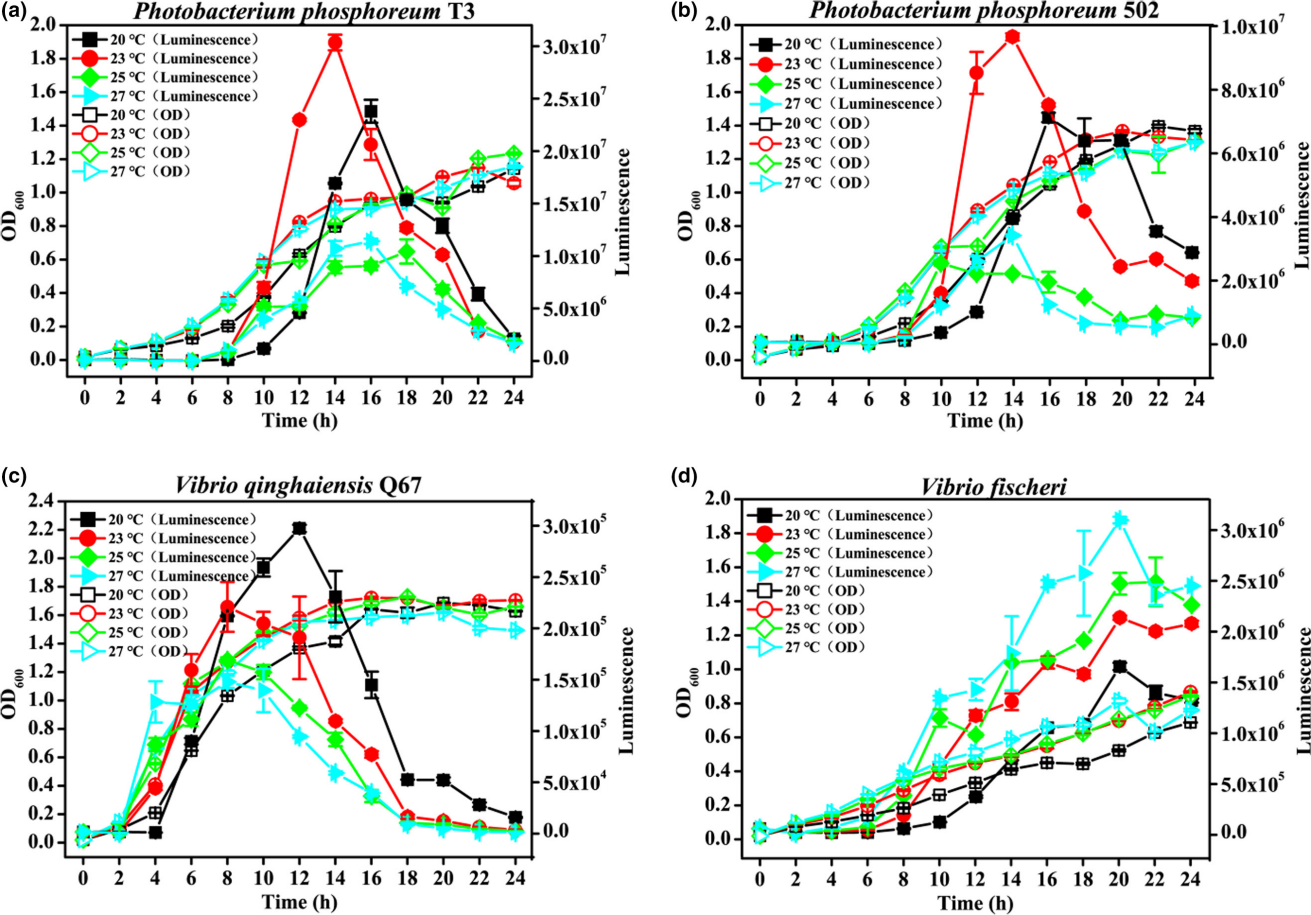
Impact of temperature on the growth and luminescence of *Photobacterium phosphoreum* T3 (a), *P. phosphoreum* 502 (b), *V. qinghaiensis* Q67 (c), and *V. fischeri* (d)

Because temperature affects the activities of enzymes that directly participate in the light emission reaction, this study investigated the impact of temperature on the growth and luminescence of four natural bioluminescence bacteria. Luminescence intensity displayed apparent differences at different temperatures but not on growth among the four strains (Figure 2). The luminescence intensity of *P*. *phosphoreum* T3 was much higher than that of the other strains. *P*. *phosphoreum* T3 and *P*. *phosphoreum* 502 showed the maximum luminescence intensity after 14‐hr cultivation at 23°C, which reached the 3.04 × 10^7^ and 9.66 × 10^6^ unit, respectively (Figure 2a,b). However, when cultured at 20°C for 12 h, *V*. *qinghaiensis* Q67 displayed the maximum luminescence intensity, which reached 2.97 × 10^5^ units (Figure 2c). For *V*. *fischeri*, higher culture temperatures exhibited higher luminescence (Figure 2d). These results indicated that the culture temperature significantly influenced the luminescence intensity, but not on growth, among the four strains. The appropriate temperature for luminescence in *P*. *phosphoreum* T3, *P*. *phosphoreum* 502, *V*. *qinghaiensis* Q67, and *V*. *fischeri* was found to be 23, 23, 20, and 27°C, respectively.

### 3.2 Preparation of bioluminescent bacteria for acute toxicity detection

The preparation methods of bioluminescent bacteria using fresh culture, suspended fresh cells, and freeze‐dried powder resulted in different acute toxicity results, while the latter exhibited stability and repeatability in toxicity detection. Indeed, the components of protectants could obviously affect cell viability and luminescence intensity. Therefore, different bacterial preparation methods and protectants used in freeze drying have been studied.

#### 3.2.1 Impact of different preparation methods of bioluminescent bacteria on the acute toxicity of contaminants

Bioluminescent bacteria were cultured and harvested according to a previous study. Subsequently, the impact of three different preparation methods on acute toxicity was investigated. Fresh culture (Method 1), fresh cells suspended in protectant solution (Method 2), and freeze‐dried powder (Method 3, here the protectant was 15% (w/v) trehalose) were prepared and subjected to acute toxicity detection. K_2_Cr_2_O_7_ and ZnSO_4_ were the standard contaminants used to detect the acute toxicity of water samples in ISO11348. Referring 20, 50% inhibition of luminescence corresponding to 3.6, 18.71 mg/L Cr (K_2_Cr_2_O_7_) and 1.08, 2.17 mg/L Zn (ZnSO_4_.7H_2_O), respectively, the EC_70_ of Cr and Zn (the contaminant concentration corresponding luminescence inhibited 70%) was calculated (28.78 and 2.9 mg/L, respectively). The acute toxicities of these contaminants were determined. The exposure time was 30 min.

**FIGURE 3:**
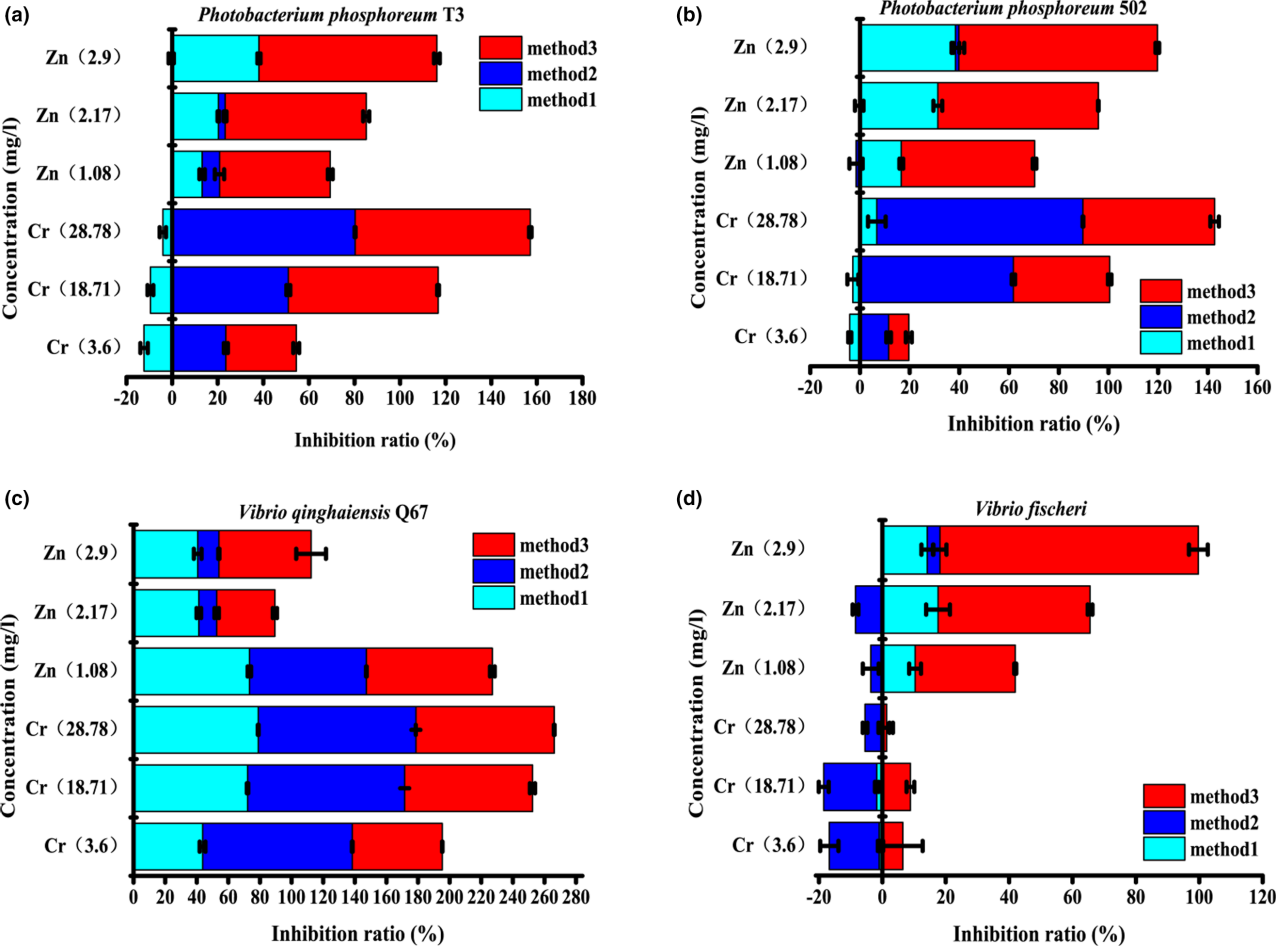
Toxic response of luminescence bacteria prepared by various approaches to chromium (Cr) and zinc (Zn). Acute toxicity of Cr and Zn to *P. phosphoreum* T3 (a), *P. phosphoreum* 502 (b), *V. fischeri* (c), and *V. qinghaiensis* Q67 (d). Method 1, Fresh culture. Method 2, Bacterial suspension. Method 3, Freeze‐dried bioluminescent bacteria. Error bars indicate the standard deviations from three independent experiments

*P*. *phosphoreum* T3 and 502 prepared by method 1 showed high luminescence inhibition with a high concentration of Zn, but not with Cr. However, the cells prepared by method 2 displayed the opposite results with Zn and Cr relative to method 1. Furthermore, the luminescent cells prepared by method 3 exhibited increased luminescence inhibition with increasing concentrations of Zn and Cr (Figure 3a,b). For *V*. *qinghaiensis* Q67, the luminescence inhibition increased as the concentration of Cr increased among the three preparation methods, whereas a high concentration of Zn showed lower luminescence inhibition (Figure 3c). Moreover, Zn showed apparent luminescence inhibition to *V*. *fischeri* prepared by method 3, and 2.17 mg/L Zn resulted in 47.98% luminescence inhibition. The inhibition value was close to 50%, as described in ISO11348, with the same concentration of Zn. However, bacteria prepared by methods 1 and 2 showed no obvious inhibition of luminescence to Zn. Nevertheless, Cr displayed no distinct difference in luminescence inhibition among the three preparation methods (Figure 3d). These results suggest that bioluminescent bacteria prepared by different approaches significantly affect the acute toxic sensitivity of cells to contaminants. Indeed, the freeze‐dried powder was the best one for the preparation of bioluminescent bacteria for acute toxicity detection.

#### 3.2.2 Protectants used in freeze‐drying process and its optimization

Protectants used in cell freeze‐drying usually include skim milk powder, trehalose, sucrose, glucose, sorbitol, arginine, glutamate, etc. (Dong et al., 2011). The skim milk powder protected cells well in freeze‐drying processes, while it clearly influenced the acute toxicity of contaminants (data not shown). Therefore, trehalose, sucrose, sorbitol, and monosodium glutamate were used to investigate their effects on cell viability and the toxicity of contaminants. The freshwater strain Q67, which exhibited lower osmotic pressure tolerance compared with other strains, was used to investigate the impact of protectants on the acute toxicity of the standard contaminant (3.6 mg/L Cr). The exposure time was 15 min. The results showed that Q67, freeze‐dried in trehalose and sucrose, exhibited a higher luminescence decrease with 3.6 mg/L Cr. In contrast, monosodium glutamate and sorbitol obviously decreased the acute toxicity of 3.6 mg/L Cr to Q67. Luminescence inhibition decreased when the sorbitol concentration exceeded 6% (w/v) (Table 1). These results indicated that trehalose, sucrose, and low concentrations of sorbitol could protect cell survival in freeze‐drying processes, whereas monosodium glutamate decreased the acute toxicity of Cr to Q67. In spite of the sorbitol decreasing acute toxicity of Cr to Q67, trehalose, sucrose, and low concentration sorbitol were suitable as protectants for this strain.

**TABLE 1:** Impact of different protectant on luminescence and luminescence inhibition. The standard contaminants and strain used in this work are 3.6 mg/L Cr and *V*. *qinghaiensis* Q67

Concentration (%, w/v)	2	4	6	8	10	12	14	16	18
Trehalose									
Luminescence × 10^5^	0.2	0.8	1.5	1.3	1.4	1.4	1.5	1.5	1.6
Inhibition ratio (%)	40	43	62	66	62	65	67	67	58
Sucrose									
Luminescence × 10^5^	0.1	0.6	1.2	1.3	1.4	1.4	1.5	1.6	1.6
Inhibition ratio (%)	40	36	48	48	47	44	42	41	42
Sorbitol									
Luminescence × 10^5^	0.4	1.2	1.8	1.5	1.6	1.6	1.2	1.1	0.7
Inhibition ratio (%)	38	21	25	15	23	16	15	17	13
Monosodium glutamate									
Luminescence × 10^5^	0.1	0.2	0.6	0.6	0.4	0.5	0.2	0.2	0.1
Inhibition ratio (%)	11	35	43	35	39	23	36	31	21

**FIGURE 4:**
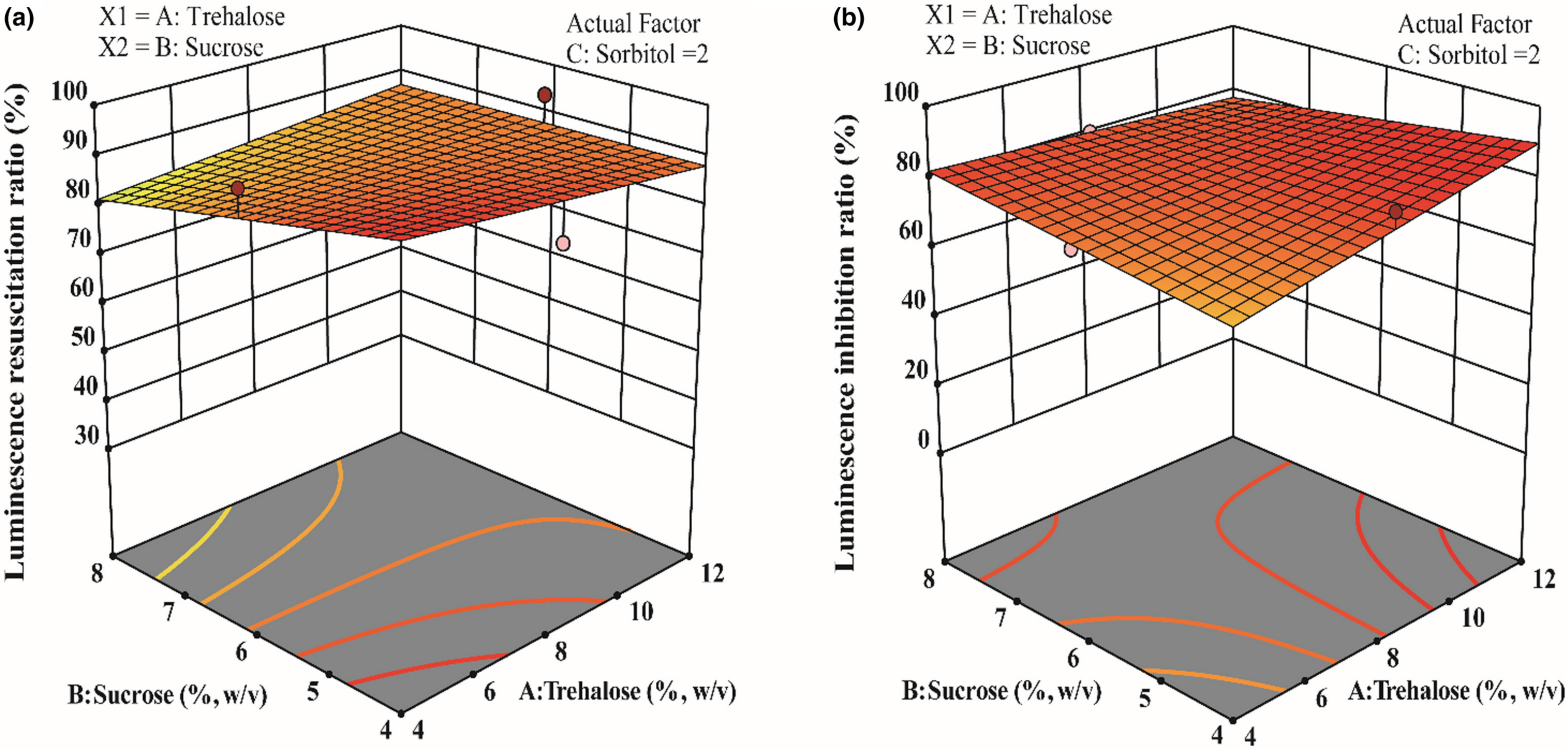
Effects of different protectants on luminescence resuscitation (a) and acute toxicity (b) were determined by response surface analysis. Concentration of sorbitol was 2% (w/v) in (a) and (b). Cr (3.6 mg/L) was used as the standard contaminant in (b)

Considering that the luminescence of these freeze‐dried bacteria with mono protectants was low after resuscitation (Table 1) and to reduce the cost of trehalose, the combined protectants were optimized using response surface analysis. Q67 protected with monosodium glutamate did not only reveal low luminescence resuscitation but also displayed a decrease in acute toxicity of Cr to this strain (Table 1). Therefore, trehalose (4%, 8%, and 12%, w/v), sucrose (4%, 6%, and 8%, w/v), and sorbitol (2%, 4%, and 6%, w/v) were used for response surface analysis to obtain the combined protectants. The 3.6 mg/L Cr was used for the luminescence inhibition test, and the exposure time was 15 min. Freeze‐dried cells showed higher luminescence resuscitation when trehalose and sucrose combined with 2% (w/v) sorbitol were used as protectants. In addition, 3.6 mg/L Cr showed high luminescence inhibition to these cells protected with combined protectants (Figure 4). Beyond expectation, the optimized protectants increased the acute toxicity of Cr to Q67. Considering the luminescence resuscitation and inhibition ratio, the software recommended the optimal combined protectants containing trehalose 12%, sucrose 4%, and sorbitol 2%. The optimal protectant was used to prepare a freeze‐dried Q67 powder. Then, acute toxicity and luminescence resuscitation measurements were conducted, and the results displayed an 80.74 ± 0.94% luminescence resuscitation ratio and 88.51 ± 0.56% luminescence inhibition ratio, which were close to the predicted values of 89.7355% and 88.0762%, respectively, by software. The lower luminescence resuscitation ratio may be due to the rapid change in temperature. However, the lower luminescence resuscitation cannot limit the application of these combined protectants for acute toxicity detection because the luminescence inhibition ratio did not exhibit obvious variation relative to the predicted value.

### 3.3 Application of freeze‐dried bioluminescent bacteria for acute toxicity detection

According to previous investigations, protectants containing 12% trehalose, 4% sucrose, and 2% sorbitol were used to prepare these four freeze‐dried natural bioluminescent bacteria cells. Then, the freeze‐dried powder of bioluminescent bacteria was used to determine the acute toxicity of the contaminants. The exposure time was 15 min.

#### 3.3.1 Acute toxicity detection of heavy metals and BPA

**FIGURE 5:**
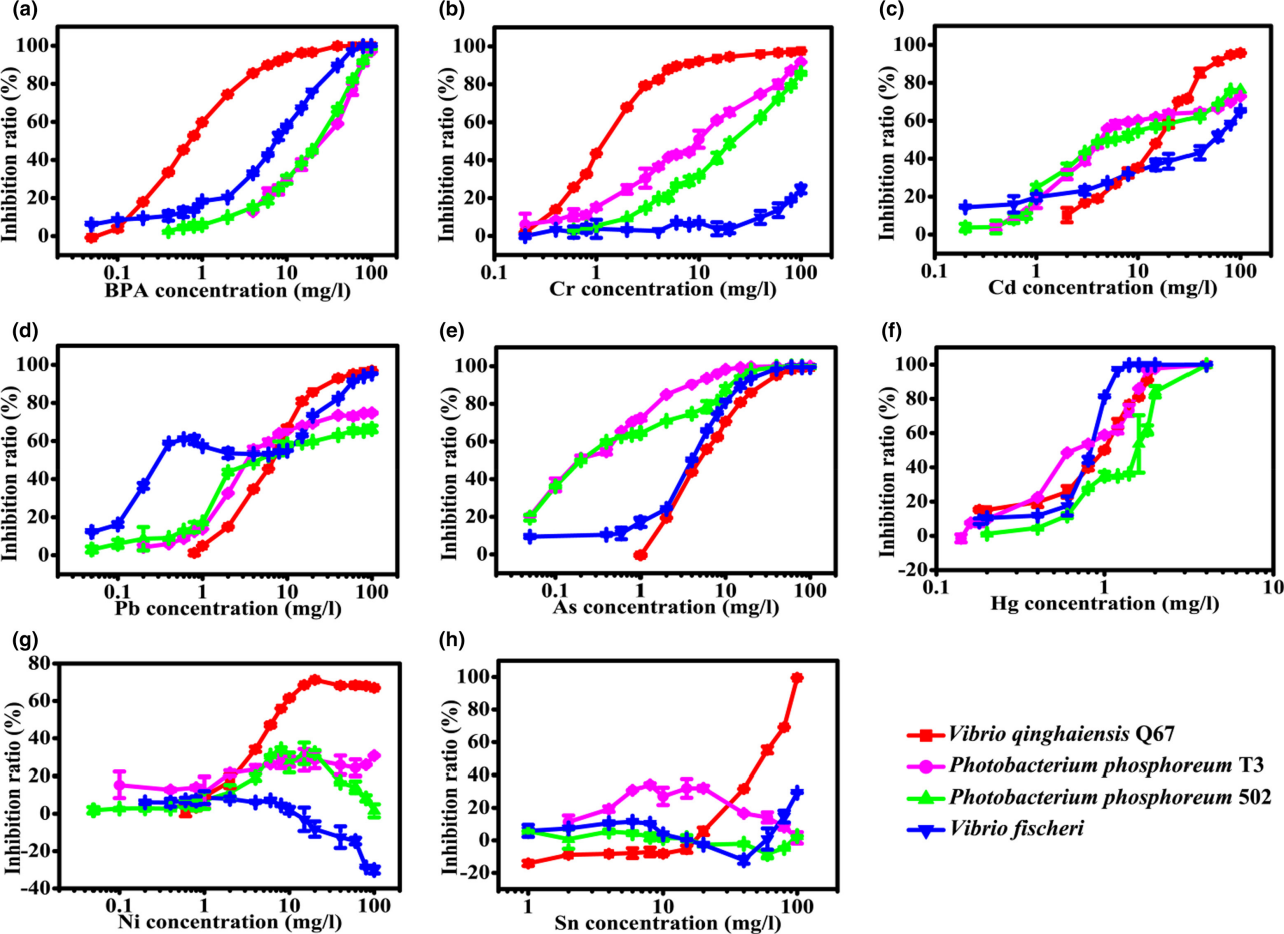
Acute toxicity of BPA (a), Cr (b), Cd (c), Pb (d), As (e), Hg (f), Ni (g), and Sn (h) to four bioluminescent strains

All contaminants used in this work exhibited obvious luminescence inhibition of *V*. *qinghaiensis* Q67 (Figure 5). A high luminescence inhibition ratio of BPA, Cr, and Hg to Q67 appeared at a low concentration of less than 2 mg/L (Figure 5a,b,f). At concentrations below 15 mg/L, Pb, As, and Ni showed strong luminescence inhibition (Figure 5d,e,g). In addition, 30 mg/L Cd and 80 mg/L Sn displayed an 80% luminescence inhibition ratio (Figure 5c,h). Both *P*. *phosphoreum* T3 and 502 strains exhibited sensitive responses to Cd, Pb, As, Hg, BPA, and Cr. Low concentrations of Cd, Pb, As, and Hg strongly inhibited the luminescence of these strains (Figure 5c–f). However, the luminescence inhibition ratio increased slowly with increasing concentrations of BPA and Cr in *P*. *phosphoreum* T3 and 502. The 20 mg/L BPA and 20 mg/L Cr inhibited the 44.24% and 65.33% luminescence of *P*. *phosphoreum* T3, respectively, while it inhibited the 44.81% and 48.93% luminescence of *P*. *phosphoreum* 502, respectively. In addition, the luminescence inhibition of Ni and Sn to *P*. *phosphoreum* T3 and 502 showed no obvious increase with increasing concentration (Figure 5g,H). These results indicated that *P*. *phosphoreum* T3 and 502 were unavailable for the toxicity detection of Ni and Sn. Consequently, the application of these strains to trace contaminants in samples is limited because their toxic response to contaminants is not sensitive. *V*. *fischeri* exhibited sensitive toxic responses to low concentrations of Hg, and 1.2 mg/L Hg inhibited 96.97% luminescence of *V*. *fischeri* (Figure 5f). In addition, low concentrations of BPA and As showed high luminescence inhibition, and 10 mg/L BPA and As inhibited 57.65 and 81.33%, respectively (Figure 5a,e). However, the luminescence inhibition slowly increased with increasing Cd concentration, and 100 mg/L Cd inhibited 65.17% luminescence (Figure 5c). Interestingly, the low concentration of Pb exhibited high luminescence inhibition, and 0.8 mg/L Pb inhibited 60.60% luminescence (Figure 5d). In addition, Ni and Sn showed no obvious luminescence inhibition to *V*. *fischeri*, and 100 mg/L Cr and Sn showed 24.59% and 29.29% luminescence inhibition, respectively (Figure 5g,h). These results suggest that the application of *V*. *fischeri* to the measurement of various contaminants is limited.

#### 3.3.2 Comparing the acute toxicity of contaminants to different bioluminescent bacteria

The acute toxicity of the contaminant was denoted by EC_50_, which was obtained by fitting the curve. The EC_50_ values of each contaminant to luminescent bacteria are listed in Table 2. Logistic, Weibull, and dose–response models were used to fit the concentration–inhibition data. The logistic model exhibited good fitting quality to all concentration–inhibition data, and the value of R^2^ ranged from 0.948 to 0.999. The EC_50_ values of each contaminant to different bioluminescent strains were compared, and a significant difference was demonstrated by statistical analysis (Table 2). The EC_50_ values of BPA, Cr, Ni, and Sn to Q67 were 0.674, 1.313, 5.941, and 54.077 mg/L, respectively. The EC_50_ of these contaminants to Q67 was much lower than that of other luminescent bacteria, and the difference was significant (Table 2). In addition, the EC_50_ of Cd to *P*. *phosphoreum* T3 and 502 was 4.162 and 5.163 mg/L, respectively, and the EC_50_ of As for these strains was 0.229 and 0.252 mg/L, respectively. Although *V*. *fischeri* showed high luminescence inhibition at lower concentrations of Pb relative to other luminescent strains, the inhibition did not increase as the concentration increased, while the others did. Furthermore, Hg showed a strong acute toxicity to all strains, and EC_50_ of *V*. *qinghaiensis* Q67, *P*. *phosphoreum* T3, *P*. *phosphoreum* 502, and *V*. *fischeri* were 0.911, 0.696, 1.358, and 0.771 mg/L, respectively. These results suggested that Q67 exhibited obvious acute toxicity to more contaminants than the other luminescent bacteria used in this study. *V*. *qinghaiensis* Q67 is the most appropriate strain for acute toxicity detection and further mixture toxicity studies because of its broad‐spectrum response to contaminants.

**TABLE 2:** Comparison of EC_50_ of contaminants to different luminescent bacteria. The EC_50_ result is shown as average ± standard error. The different letters a, b, c, and d indicate that the difference is statistically significant, but the same letter is considered not significant

Contaminant	*Vibrio qinghaiensis* Q67 (mg/l)	*Photobacterium phosphoreum* T3 (mg/l)	*Photobacterium phosphoreum* 502 (mg/l)	*Vibrio fischeri* (mg/l)
BPA	0.674 ± 0.003^c^	23.181 ± 0.003^a^	21.936 ± 0.518^a^	7.295 ± 0.016^b^
Cr	1.313 ± 0.008^c^	8.608 ± 0.146^b^	20.936 ± 0.154^a^	/
Cd	11.137 ± 0.162^b^	4.162 ± 0.082^d^	5.634 ± 0.168^c^	46.827 ± 1.529^a^
Pb	5.921 ± 0.043^a^	3.488 ± 0.107^c^	3.937 ± 0.005^b^	/
As	4.674 ± 0.013^a^	0.229 ± 0.008^d^	0.252 ± 0.002^c^	3.851 ± 0.017^b^
Hg	0.911 ± 0.005^b^	0.696 ± 0.008^d^	1.358 ± 0.043^a^	0.771 ± 0.009^c^
Ni	5.941 ± 0.044	/	/	/
Sn	54.077 ± 0.479	/	/	/

## 4 DISCUSSION

Bisphenol A and heavy metals are not only widespread in the environment but are also limited in food. Current standard chemical analysis methods and conventional bioassays for contaminant detection have some disadvantages, such as time consumption, high cost, and poor repeatability, and they fail to reveal the biotoxicity of contaminants. Hence, rapid acute toxicity detection of contaminants in foods or environments using bioluminescent bacteria could be widely applied. Nonetheless, the influence of complex ingredients in food on target contaminants was inevitable; thus, the bioluminescent bacteria detected whole mixture toxicity of food. It could be used as a fast‐screening sensor for food safety assessment before using high‐cost standard methods (Alocija & McLean, 2007). Indeed, this was based on the fact that bioluminescent bacteria exhibit a toxic response to more contaminants. However, the contaminants in the food were trace and varied. Therefore, a bioluminescent bacterial strain that responds sensitively to various contaminants limited in food should be investigated. In this study, the results indicated that freeze‐dried *V. qinghaiensis* Q67 was the best one to quickly screen the total toxicity of various contaminants in food (Figure 5 and Table 2). However, the application of Q67 in toxicity fast screening for foods or others needs to be validated by further studies.

According to the previous study, culture conditions affected the growth and luminescence of these bioluminescent bacteria. In this study, an obvious difference in luminescence was not observed when the shaking rate increased. In addition, the extracellular medium pH showed less influence on luminescence of Q67 (Ma et al., 1999), and the other strains exhibited a narrow pH tolerance ranging from 6.5 to 7.5 (Bulich & Isenberg, 1981; Tarkpea et al., 1986). Similar results were demonstrated by Q67 and the other strains in this study. Light emission is the product of an enzymatic reaction, and the temperature directly affects the activity of luciferase and fatty acid reductase. The impact of temperature on cell growth and luminescence was studied, and the results suggested that temperature significantly influences the luminescence of these four strains. Therefore, many studies on *lux* operon cloning (especially in *Escherichia coli*) have used the *luxCDABE* operon of *Photobacterium luminescens* because this strain was isolated from a human wound, resulting in strong luminescence at 37°C (Ivask et al., 2009; Kurvet et al., 2011). However, the application of acute toxicity detection using bioluminescent bacteria is commonly performed at room temperature. Consequently, strains exhibiting strong luminescence at room temperature are a priority for acute toxicity detection. Based on the results above, the luminescence of bioluminescent bacteria was mainly affected by temperature but not dissolved oxygen and extracellular pH because the activity of enzymes catalyzing the light emission reaction was directly influenced by temperature and intracellular pH. The appropriate temperature for luminescence of these four strains displayed differences, but all were close to room temperature.

The effects of different bioluminescent bacterial preparation methods on acute toxicity were investigated. Previous studies have used fresh culture (method 1) to detect the acute and chronic toxicity of contaminants (Froehner et al., 2002; Zhu et al., 2009). ISO11348 introduced method 2 (fresh cells suspended in protectant solution) and method 3 (freeze‐dried powder) for acute toxicity measurements in water. In this study, the results suggested that the freeze‐dried bioluminescent cells displayed a strong toxic response to contaminants. Indeed, the freeze‐dried powder could retain the activity of cells for a long time, and the same batch of cultured cells might eliminate the error from different physiological luminescent cells (Dong et al., 2011). Furthermore, the components of protectants used in freeze‐drying processes were optimized, and the results indicated that freeze‐dried Q67 prepared with protectants containing 12% trehalose, 4% sucrose, and 2% sorbitol exhibited a strong luminescence inhibition response to standard contaminants and high luminescence resuscitation. The acute toxicity of standard contaminants (Cr) to freeze‐dried Q67 was much higher than expected (Figure 4).

Considering the lower osmotic pressure tolerance of *V. qinghaiensis* Q67 compared to other strains, this work used Q67 to optimize the protectant for freeze‐drying processes by response surface analysis. In fact, application of the protectant on *P*. *phosphoreum* T3, *P*. *phosphoreum* 502, and *V*. *fischeri* was reasonable because 3.6 mg/L Cr displayed higher luminescence inhibition to bioluminescent cells prepared with this optimized protectant than with 15% trehalose (Figures 2 and 5). Although the toxicity of BPA in degradation was investigated using *V*. *fischeri* (Han et al., 2015), the strong acute toxicity of BPA to Q67 has barely been studied. Furthermore, the acute toxicity of six heavy metals (Hg, Cu, Zn, Cd, Ni, and Mn) to *P*. *phosphoreum* T3 and *V*. *qinghaiensis* Q67 was compared in a previous study (Ma et al., 1999). The results showed that the EC_50_ of Cd and Ni to T3 was much higher than that of Q67 but reversed to Hg, which was similar to the results of this study (Table 2). Moreover, acute toxicity of heavy metals to *V*. *fischeri* was investigated (Abbas et al., 2018), and the results showed some differences relative to this study. Beyond expectation, 0.8 mg/L Pb inhibited 60.60% of luminescence, but a decrease over this concentration (Figure 5c), which might be explained by hormesis (Shen et al., 2009; Zheng et al., 2017). All results of acute toxicity tests for these four bioluminescent bacteria were different from other studies (Gérald Thouand & Marks, 2014). Nevertheless, this study focused on comparing the acute toxicity of contaminants to these four bioluminescent bacteria, which were performed under the same preparation, exposure, and detection conditions. These results indicate that the most appropriate bioluminescent bacteria strain to detect acute toxicity of contaminants used in this study was Q67. The mixture toxicity of BPA and heavy metals or other contaminants was investigated using Q67 in both the environment and food safety fields. This is a rapid and low‐cost approach compared with animal experiments, and not against the 3R principles regarding the use of animals (Chen et al. (2021); Riebeling et al. (2018); and Su et al. (2018)).

## 5 CONCLUSION

The results of this study validated that the differences in the acute toxicity of contaminants to *P. phosphoreum* T3, *V. fischeri*, *P. phosphoreum* 502, and *V. qinghaiensis* Q67 were statistically significant (Table 2). Moreover, we found that Q67 exhibited acute toxic responses to more contaminants than the other strains. BPA exhibited the strongest acute toxicity to Q67 (EC_50_ = 0.674 mg/L) compared to the other bioluminescent bacterial strains. Compared to other strains, *V. qinghaiensis* Q67 was suggested to be the better strain to be used as a potential biosensor for non‐specific contaminants to facilitate the rapid screening and assessment of acute toxicity of samples.

## CONFLICT OF INTEREST

The authors declare that they do not have any conflict of interest.

## ETHICAL APPROVAL

This study does not involve any human or animal testing.

## INFORMED CONSENT

Written informed consent was obtained from all study participants.

## Data Availability

Data available on request from the authors.
